# Supplementary material for: A trafficome-wide RNAi screen reveals deployment of early and late secretory host proteins and the entire late endo-/lysosomal vesicle fusion machinery by intracellular Salmonella
Source: PLoS Pathog. 2020 Jul 13;16(7):e1008220. doi: 10.1371/journal.ppat.1008220 (PMC7377517; doi:10.1371/journal.ppat.1008220)
Supplement: S1 Text — (DOCX) [file ppat.1008220.s001.docx]

## Suppl. Text

## Considerations for screen design and setup

#### The setup of an LCI-based RNAi screen required several controls, and technical and biological prerequisites that are summarized below.

**Table.** Different types of general requirements and corresponding consequences an RNAi screen which applies LCI for the identification of host factors involved in SIF formation by intracellular *Salmonella* has to fulfill.

| Type of prerequisite | | Prerequisite | | Necessity/Solution |
| --- | --- | --- | --- | --- |
| biological | label for structure of interest*Salmonella* controls for structure of interest | | LAMP1 as marker of SIFWT: SIF-positive*ssaV*: SIF-negative | |
| technical | fast and gentle microscopic systemmultiwell-compatible 37 °C incubationmultiwell-compatible sufficient magnificationautofocus systemautofocus-compatible multiwell plates | | spinning disk confocal microscope (Zeiss)box incubation (custom-made)LD 40x/0.6 objective (+ bottom thickness correction collar, Zeiss)infrared-based system (Definite Focus, Zeiss)96-well Clear Bottom Black Cell Culture Microplates (0.5 mm bottom, Corning) | |
| siRNA-related | harmlessness of transfection reagentharmlessness of siRNA as reagentgeneral efficacy of siRNAphenotype-interfering siRNAcontrol of phenotype-interfering siRNA | | mock transfection controlscrambled AllStars siRNA controllethal PLK1 siRNA controlSIF-abolishing SKIP siRNART-PCR and WB of SKIP knockdown | |
| data analysis | primary analysiscollection of analyzed data | | manual visual inspectionSifScreen utility | |

#### i) A microscopy system capable of fast image acquisition and an illumination regime only causing minimal photodamage. While use of wide-field microscopy (WFM) is straightforward, these systems lack high resolution, at least without deconvolution. This point is crucial in our setup focusing on SIF as fine tubular structures. In contrast, confocal laser-scanning microscopy (CLSM) provides high resolution, but also imposes high local light doses increasing photo-damage. As summarized below, a system perfectly matching our requirements is SDCM. It provides high resolution by confocality, speed by focused simultaneous illumination of the complete field of view, and therefore gentleness due to the reduced need of excitation. Speed is additionally increased in our system by using a 512 x 512 pixel camera (compared to 1,024 x 1,024 pixel or even higher resolutions often used).

**Table**. Characteristics of microscopy systems concerning LCI and RNAi screening.

| System | | Advantage | | Disadvantage | |
| --- | --- | --- | --- | --- | --- |
| WFM | | fastgentle to cells | | lowest resolution (without deconvolution) | |
| CLSM | highest resolution | | slowhigh local illumination, photo-damage | |  |
| SDCM | high resolutionfastgentle to cells | | lower resolution as CLSM (due to camera) | |  |

#### ii) LCI requires incubation at 37 °C in a multi-well compatible format for the RNAi screen. Though stage top solutions exist for multi-well plate incubation, the solution of choice is a box incubation enclosing the entire microscope body. This guarantees uniform heating of all relevant microscope parts, thus, reducing thermal drift. We used a custom-made incubation chamber.

#### iii) In previous RNAi screens investigating *Salmonella* or other intracellular pathogens, the analysis was performed by recording signals of the pathogen from a channel separate from the cellular signal, such as cell shape, nucleus, etc. Though here we also had the *Salmonella* signal separate from the cell (mCherry-labelled vs. eGFP-labelled, see Figure 1A), the signal/structure of interest were SIF, i.e. represented by a cellular marker. As SIF are tenuous tubules of less than 200 nm diameter, imaging with low magnification objectives such as 10x or 20x usually used for RNAi screens is inappropriate. High magnification objectives such as 63x or 100x, albeit best for SIF identification, are likewise inappropriate due to insufficient numbers of cells per field of view for proper quantitative analysis. Thus, we decided to use a 40x objective. Multi-well plates excluded the use of high numerical aperture 40x objectives that demand immersion media. This resulted in the selection of a long working distance (LD) 40x/0.6 NA objective (Zeiss, with bottom thickness correction collar).

#### iv) The requirement to use multi-well plates to cope with a large number of different siRNAs/conditions in an RNAi screen necessitates automated microscopy. This not only means an automated stage but, much more significant, some kind of focus strategy or autofocus. Most customary sophisticated microscopic systems or their software offer the possibility of software autofocus. A major drawback about LCI with this solution is not only the use of the actual acquisition laser, thus exposing the samples to a potentially harmful, yet basically superfluous light/energy source, but also the concomitant loss of speed. Thus, we used an infrared-based autofocus system (IRF, from Zeiss called ‘Definite Focus’). The IRF measures the distance between objective and sample bottom with a non-invasive LED and corrects this distance if necessary. Using low magnification objectives this system is perfectly feasible.

#### Because our screen required the use of 40x objective, we encountered a major obstacle: standard cell culture multi-well plates with plastic bottom showed a high degree of variation in bottom thickness (ca. 1 mm). This variation prohibited IRF, at least with the 40x objective used. None of the standard multiwell plates from various manufacturers provided sufficiently consistent thickness. Next, we tested glass-bottom plates (ca. 0.17 mm thickness), by design providing the lowest degree of variation. Unexpectedly, independent of the manufacturer this type of plates again did not allow to screen a full plate without loss of focus despite the application of IRF. The use of plates with glass-like synthetic bottoms (e.g. based on cyclic olefin such as Greiner Screenstar plates) also did not work. Since we could not fully explain these difficulties except the fact that the specific 40x objective we were using was not intended for screening applications, we tested other types of plates. Finally, we found 96-well Clear Bottom Black Cell Culture Microplates with an atypical 0.5 mm bottom thickness fulfilling all our needs as summarized below.

**Table**. Suitability of various types of multi-well plates for screening with an LD 40x objective and IRF.

| Type | Bottom thickness in mm | | Applicability | | Representative suppliers | |
| --- | --- | --- | --- | --- | --- | --- |
| standard plastic | 1 | no, bottom variation too high | | Corning, Greiner, Thermo/Nunc, TPP | |  |
| glass | 0.17 | no, otherwise incompatible | | Corning, Greiner, MatTek, Thermo/Nunc | |  |
| cyclic olefin/synthetic | 0.19 | no, otherwise incompatible | | Corning, Greiner | |  |
| proprietary specialized plastic | 0.5 | yes | | Corning | |  |

#### Using these plates, we were able to define that the acquisition of a complete plate delivers satisfying results regarding the focus of cells and SIF if the focus is adjusted to the plane where most bacteria are located intracellularly 1 h p.i.

#### v) We next defined the timeframe of LCI. In pilot experiments, we determined eight positions per well to represent a feasible number for completion of one round of acquisition within 1 h if a 96-well plate is scanned/acquired with single bacteria-focused Z-planes. This number of positions covers a sufficient number of cells/events for quantitative analyses. Acquisition in 1 h intervals until 8 h p.i allows to fully cover the long-term dynamics of SIF. Short-term dynamics [1] cannot be covered this way. The time required to register signals in 96-well plates in 1 h intervals excluded the acquisition of Z-stacks.

#### vi) LCI of endosomal remodeling requires fluorescent labeling of the endosomal compartment. Fluid phase markers may be used for labeling [2], however, this would introduce additional incubation steps especially colliding with siRNA transfection. Alternatively, tagging of the late endosomal/lysosomal membrane protein LAMP1 can serve as a marker for SIF formation. We used a HeLa cell line generated by lentiviral transfection for constitutive expression of LAMP1-GFP [3].

#### vii) We established that 5,000 cells per well in a 96-well plate seeded 72 h before infection yields a suitable cell density. This number yields sufficiently dense, yet not too dispersed cell populations applicable to treatment with siRNAs in such a concentration. Of central importance in maintaining this population density was the use of cell culture medium containing inactivated fetal calf serum (iFCS) for washing steps during the infection process. We observed that washing with phosphate-buffered saline (PBS) resulted in loss of a high number of cells.

#### viii) We established that a multiplicity of infection (MOI) of 15 with synchronization of infection by centrifugation yielded sufficiently high numbers of infected cells. These parameters avoided too much cytosolic hyper-replication, a phenomenon in which cytosolic *Salmonella* start to excessively replicate independent of the strain background knodler [4].

#### For data collection, the SifScreen utility allowed the entry of following information:

#### i) Selection of wells to be analyzed, or wells that were filled, respectively, in a 96-well format by activating/deactivating individual wells through double-clicking, i.e. determining which wells will appear in the results report (see (vi) below).

#### ii) Selection of any of the individual positions for each well being acquired, i.e. determining for which position subsequent data input described in (iii)-(v) applies.

#### iii) Three tick boxes sampling SIF formation can be checked depending on each other, i.e. each latter one is only activated if the respective previous one is selected (ticking ‘SIF?’ is only possible if ‘Salmonella Infection?’ was ticked, ticking ‘Salmonella Infection’ is only possible if ’Cells visible?’ was ticked). The outcome of these tick boxes appears in the results report in a binary format (1 for ticked, 0 for unticked) to allow for easier quantification and summarization of overall results.

#### iv) Ten tick boxes sampling a variety of other phenotypes can be checked appearing in the results report as plain text if selected. On the one hand, this comprises phenomena affecting the results in general (‘Out of Focus?’, ‘One/few Cells?’, ‘Cells sick/dying?’). This allows the exclusion of positions/wells from analysis or at least to take that into account to avoid biased results. On the other hand, this encompasses atypical phenotypes concerning SMM (rest of boxes) not to lose this information even if it was not the primary goal of this screen.

#### v) A ‘Notes’ box allows entering any text, e.g. for phenomena/phenotypes not covered by (iv), also appearing in the results report as entered in this field.

#### vi) By clicking ‘Save Results’ a results report is created in an Excel format.

## References

1. Kehl A, Hensel M. Live cell imaging of intracellular Salmonella enterica. Methods Mol Biol. 2015;1225:199-225. doi: 10.1007/978-1-4939-1625-2_13. PubMed PMID: 25253257.

2. Zhang Y, Hensel M. Evaluation of nanoparticles as endocytic tracers in cellular microbiology. Nanoscale. 2013;5(19):9296-309. doi: 10.1039/c3nr01550e. PubMed PMID: 23942623.

3. Krieger V, Liebl D, Zhang Y, Rajashekar R, Chlanda P, Giesker K, et al. Reorganization of the endosomal system in *Salmonella*-infected cells: the ultrastructure of *Salmonella*-induced tubular compartments. PLoS Pathog. 2014;10(9):e1004374. doi: 10.1371/journal.ppat.1004374. PubMed PMID: 25254663.

4. Knodler LA, Nair V, Steele-Mortimer O. Quantitative assessment of cytosolic *Salmonella* in epithelial cells. PLoS One. 2014;9(1):e84681. doi: 10.1371/journal.pone.0084681. PubMed PMID: 24400108; PubMed Central PMCID: PMC3882239.
