# Supplementary material for: A trafficome-wide RNAi screen reveals deployment of early and late secretory host proteins and the entire late endo-/lysosomal vesicle fusion machinery by intracellular Salmonella
Source: PLoS Pathog. 2020 Jul 13;16(7):e1008220. doi: 10.1371/journal.ppat.1008220 (PMC7377517; doi:10.1371/journal.ppat.1008220)
Supplement: S3 Table — (DOCX) [file ppat.1008220.s004.docx]

| **Designation** | **Relevant genotype** | | **Source/Reference** | |  |  |
| --- | --- | --- | --- | --- | --- | --- |
| **STM strains** | | | | | |  |
| NCTC 12023 | | wild type | | lab collection | | |
| P2D6 | | *ssaV*::mTn*5* | | [1] | | |
| MvP1897 | | Δ*phoN*::P_EM7_::sfGFP *aph* | | This study | | |
| **Plasmids** | | | | | |  |
| p3099 | P_CMV_::eGFP::RAB8A | | This study | |  |  |
| p3101 | P_CMV_::eGFP::RAB9A | | This study | |  |  |
| p3451 | P_CMV_::hLAMP1::mCherry | | [2] | |  |  |
| p4080 | P_CMV_::mRuby2-C1 | | This study | |  |  |
| p4081 | P_CMV_::mRuby2-N1 | | This study | |  |  |
| p4094 | P_CMV_::mRuby2::STX8 | | This study | |  |  |
| p4122 | P_CMV_::mRuby2::RAB1B | | This study | |  |  |
| p4209 | P_CMV_::mRuby2::RAB8B | | This study | |  |  |
| p4210 | P_CMV_::mRuby2::RAB3A | | This study | |  |  |
| p4232 | P_CMV_::mRuby2::CLTA | | This study | |  |  |
| p4250 | P_CMV_::mRuby::RAB1A | | This study | |  |  |
| pcDNA3-mRuby2 | P_CMV_::mRuby2 | | Addgene (#40260), [3] | |  |  |
| pEGFP VAMP3 | P_CMV_::eGFP::VAMP3 | | Thierry Galli, Paris, Addgene (#42310), [4] | |  |  |
| pEGFP VAMP7 | P_CMV_::eGFP::VAMP7 | | Thierry Galli, Paris, Addgene (#42316), [5] | |  |  |
| pEGFP VAMP8 | P_CMV_::eGFP::VAMP8 | | Thierry Galli, Paris, Addgene (#42311), [4] | |  |  |
| pEGFP-C1 | P_CMV_::EGFP-C1 | | Clontech | |  |  |
| pEGFP-C3 | P_CMV_::EGFP-C3 | | Clontech | |  |  |
| pEGFP-N1 | P_CMV_::EGFP-N1 | | Clontech | |  |  |
| pEGFP-Vamp4 | P_CMV_::VAMP4::eGFP | | Wanjin Hong, Singapur | |  |  |
| pENTR223_RAB1A | RAB1A | | DNASU GTPases (HsCD00509534) | |  |  |
| pENTR223_RAB1B | RAB1B | | DNASU GTPases (HsCD00509534) | |  |  |
| pENTR223_RAB3A | RAB3A | | DNASU GTPases (HsCD00507538) | |  |  |
| pENTR223_RAB8B | RAB8B | | DNASU GTPases (HsCD00288320) | |  |  |
| pENTR223_STX8 | STX8 | | DNASU 16967 (HsCD00507664) | |  |  |
| pFPV-mCherry | P*_rpsM_*::mCherry | | Addgene (#20956), [6] | |  |  |
| pGL-Rab7 wt | P_SV40_::RAB7::eGFP | | Martin Aepfelbacher, Hamburg | |  |  |
| pLenti Vamp2 pHtomato | VAMP2::pHtomato | | Yulong Li, Beijing | |  |  |
| pMRXIP GFP-Stx7 | P_CMV_::GFP::STX7 | | Addgene (#45921), [7] | |  |  |
| pMRXIP GFP-Vti1b | P_CMV_::GFP::VTI1B | | Addgene (#45922), [7] | |  |  |

### **References**

1. Shea JE, Hensel M, Gleeson C, Holden DW. Identification of a virulence locus encoding a second type III secretion system in *Salmonella typhimurium*. Proc Natl Acad Sci U S A. 1996;93(6):2593-7.

2. Krieger V, Liebl D, Zhang Y, Rajashekar R, Chlanda P, Giesker K, et al. Reorganization of the endosomal system in *Salmonella*-infected cells: the ultrastructure of *Salmonella*-induced tubular compartments. PLoS Pathog. 2014;10(9):e1004374. doi: 10.1371/journal.ppat.1004374. PubMed PMID: 25254663; PubMed Central PMCID: PMCPMC4177991.

3. Lam AJ, St-Pierre F, Gong Y, Marshall JD, Cranfill PJ, Baird MA, et al. Improving FRET dynamic range with bright green and red fluorescent proteins. Nat Methods. 2012;9(10):1005-12. doi: 10.1038/nmeth.2171. PubMed PMID: 22961245; PubMed Central PMCID: PMCPMC3461113.

4. Paumet F, Le Mao J, Martin S, Galli T, David B, Blank U, et al. Soluble NSF attachment protein receptors (SNAREs) in RBL-2H3 mast cells: functional role of syntaxin 4 in exocytosis and identification of a vesicle-associated membrane protein 8-containing secretory compartment. J Immunol. 2000;164(11):5850-7. doi: 10.4049/jimmunol.164.11.5850. PubMed PMID: 10820264.

5. Martinez-Arca S, Alberts P, Zahraoui A, Louvard D, Galli T. Role of tetanus neurotoxin insensitive vesicle-associated membrane protein (TI-VAMP) in vesicular transport mediating neurite outgrowth. J Cell Biol. 2000;149(4):889-900. doi: 10.1083/jcb.149.4.889. PubMed PMID: 10811829; PubMed Central PMCID: PMCPMC2174569.

6. Drecktrah D, Levine-Wilkinson S, Dam T, Winfree S, Knodler LA, Schroer TA, et al. Dynamic behavior of *Salmonella*-induced membrane tubules in epithelial cells. Traffic. 2008;9(12):2117-29. Epub 2008/09/13. doi: TRA830 [pii]

10.1111/j.1600-0854.2008.00830.x. PubMed PMID: 18785994.

7. Itakura E, Kishi-Itakura C, Mizushima N. The hairpin-type tail-anchored SNARE syntaxin 17 targets to autophagosomes for fusion with endosomes/lysosomes. Cell. 2012;151(6):1256-69. doi: 10.1016/j.cell.2012.11.001. PubMed PMID: 23217709.
