# Supplementary material for: A trafficome-wide RNAi screen reveals deployment of early and late secretory host proteins and the entire late endo-/lysosomal vesicle fusion machinery by intracellular Salmonella
Source: PLoS Pathog. 2020 Jul 13;16(7):e1008220. doi: 10.1371/journal.ppat.1008220 (PMC7377517; doi:10.1371/journal.ppat.1008220)
Supplement: S4 Table — (DOCX) [file ppat.1008220.s005.docx]

| Gene symbol | siRNA name | Catalog no. | Target sequence |
| --- | --- | --- | --- |
| --- | AllStars | SI03650318 | proprietary |
| HGS | Hs_HGS_6 | SI02659650 | GCACGTCTTTCCAGAATTCAA |
| PLK1 | Hs_PLK1_7 | SI02223844 | CGCGGGCAAGATTGTGCCTAA |
| RAB1A | Hs_RAB1A_9 | SI02662716 | AACTATAGAGTTAGACGGGAA |
| RAB7A | Hs_RAB7A_2 | SI00066395 | TCCCGTTAGATCAGCATTCTA |
| RAB11A | Hs_RAB11A_7 | SI02663206 | AAGAGCGATATCGAGCTATAA |
| SKIP/  PLEKHM2 | custom | custom | AAAACGAAGAGCAGCTGTTCA |
| STX5 | Hs_STX5A_4 | SI00048636 | CAGTGGAAATTGAAGAGCTAA |
| STX7 | Hs_STX7_7 | SI03064159 | CAGAGGATGACCTCCGTCTTA |
| VAMP7 | Hs_SYBL1_7 | SI04212453 | TAGGGCAATCGTGTCGCTAAT |
| VAMP8 | Hs_VAMP8_2 | SI02652993 | CCGACTAGGCGAATTCACTTA |
| VCP | Hs_VCP_7 | SI03019730 | AACAGCCATTCTCAAACAGAA |
| VPS11 | Hs_VPS11_6 | SI02778167 | CAGCAATATATCCGAACCATT |
