# Supplementary material for: A trafficome-wide RNAi screen reveals deployment of early and late secretory host proteins and the entire late endo-/lysosomal vesicle fusion machinery by intracellular Salmonella
Source: PLoS Pathog. 2020 Jul 13;16(7):e1008220. doi: 10.1371/journal.ppat.1008220 (PMC7377517; doi:10.1371/journal.ppat.1008220)
Supplement: S5 Table — (DOCX) [file ppat.1008220.s006.docx]

| Targeted gene | Designation | Sequence 5’-3’ |
| --- | --- | --- |
| Used for RT-PCR | | |
| GAPDH | Hs-GAPDH-qPCR-For2  Hs-GAPDH-qPCR-Rev2 | TGCACCACCAACTGCTTAGC  GGCATGGACTGTGGTCATGAG |
| HGS | Hs-HGS-qPCR-For  Hs-HGS-qPCR-Rev | CTCCTGTTGGAGACAGATTGGG  GTGTGGGTTCTTGTCGTTGAC |
| RAB1A | Hs-RAB1A-qPCR-For  Hs-RAB1A-qPCR-Rev | AGATTAAAAAGCGAATGGGTCCC  GCTTGACTGGAGTGCTCTGAAT |
| RAB7A | Hs-RAB7A-qPCR-For  Hs-RAB7A-qPCR-Rev | TACAAAGCCACAATAGGAGCTG  GCAGTCTGCACCTCTGTAGAAG |
| RAB11A | Hs-RAB11A-qPCR-For  Hs-RAB11A-qPCR-Rev | CAACAAGAAGCATCCAGGTTGA  GCACCTACAGCTCCACGATAAT |
| SKIP/  PLEKHM2 | Hs-SKIP-qPCR-For  Hs-SKIP-qPCR-Rev | TAGAGTTCATTCGTTTCGAGCTG  AAGGCGGTCTTCAAAGTCCAG |
| STX5 | Hs-STX5-qPCR-For  Hs-STX5-qPCR-Rev | AAAGCGCAAGTCCCTCTTTGA  TGAGCAATTTGTTTGTTGAGGC |
| STX7 | Hs-STX7-qPCR-For  Hs-STX7-qPCR-Rev | GGCCCAGAGGATCTCTTCTAA  ACTGTTGCCTCAATTCAGGTG |
| VAMP7 | Hs-VAMP7-qPCR-For  Hs-VAMP7-qPCR-Rev | GAGGTTCCAGACTACTTACGGT  GACACTTGAGAACTCGCTATTCA |
| VAMP8 | Hs-VAMP8-qPCR-For  Hs-VAMP8-qPCR-Rev | TGTGCGGAACCTGCAAAGT  CTTCTGCGATGTCGTCTTGAA |
| VCP | Hs-VCP-qPCR-For  Hs-VCP-qPCR-Rev | CAAACAGAAGAACCGTCCCAA  TCACCTCGGAACAACTGCAAT |
| VPS11 | Hs-VPS11-qPCR-For  Hs-VPS11-qPCR-Rev | CAAGCCTACAAACTACGGGTG  GAGTGCAGAGTGGATTGCCA |
| Used for plasmid construction | | |
| EGFP | Vf-pEGFP-C1  Vr-pEGFP-C1 | TCCGGACTCAGATCTCGAGCTCA  GGTGGCGACCGGTAGCGC |
| EGFP | Vf-pEGFP-N1  Vr-pEGFP-N1 | AGTAAAGCGGCCGCGACT  GGTGGCGACCGGTGGATC |
| RAB1A | 1f-Rab1A  1r-Rab1A | CTTCGAATTCTGCAGTCGACATGTCCAGCATGAATCCCGAATA  TCTAGATCCGGTGGATCCTCAGCAGCAACCTCCACCTGAC |
| RAB1B | 1f-Rab1B  1r-Rab1B | CTTCGAATTCTGCAGTCGACATGAACCCCGAATATGACTACCTGTTT  TCTAGATCCGGTGGATCCTCAGCAACAGCCACCGCCAGC |
| RAB3A | 1f-Rab3A  1r-Rab3A | CTTCGAATTCTGCAGTCGACATGGCATCCGCCACAGACTC  TCTAGATCCGGTGGATCCTCAGCAGGCGCAGTCCTGGTG |
| RAB8B | 1f-Rab8B  1r-Rab8B | CTTCGAATTCTGCAGTCGACATGGCGAAGACGTACGAT  TCTAGATCCGGTGGATCCTCAGCAAAGTAGCGAGCAACG |
| mRuby2 | 1f-pcDNA3-mRu-C1  1r-pcDNA3-mRu-C1 | GCGCTACCGGTCGCCACCATGGTGTCTAAGGGCGAAGAG  TCGAGATCTGAGTCCGGACTTGTACAGCTCGTCCATC |
| mRuby2 | 1f-pcDNA3-mRu-N1  1r-pcDNA3-mRu-N1 | GATCCACCGGTCGCCACCATGGTGTCTAAGGGCGAAGAG  AGTCGCGGCCGCTTTACTTTACTTGTACAGCTCGTCCATC |
| mRuby2 | Vf-pmRuby2-C1  Vr-pmRuby2-C1 | GGATCCACCGGATCTAGATAAC  GTCGACTGCAGAATTCGAAG |
| mRuby2 | Vf-pmRuby2-N1  Vr-pmRuby2-N1 | CCGCGGGCCCGGGATCCA  CTGCAGAATTCGAAGCTTGAGCTCGAGA |
| STX8 | 1f-STX8-C1  1r-STX8-C1 | CTTCGAATTCTGCAGTCGACATGGCACCGGACCCCTGGT  GTTATCTAGATCCGGTGGATCCGTTGGTCGGCCAGACTGCAA |
