# Supplementary figures and images for: A trafficome-wide RNAi screen reveals deployment of early and late secretory host proteins and the entire late endo-/lysosomal vesicle fusion machinery by intracellular Salmonella
Source: PLoS Pathog. 2020 Jul 13;16(7):e1008220. doi: 10.1371/journal.ppat.1008220 (PMC7377517; doi:10.1371/journal.ppat.1008220)

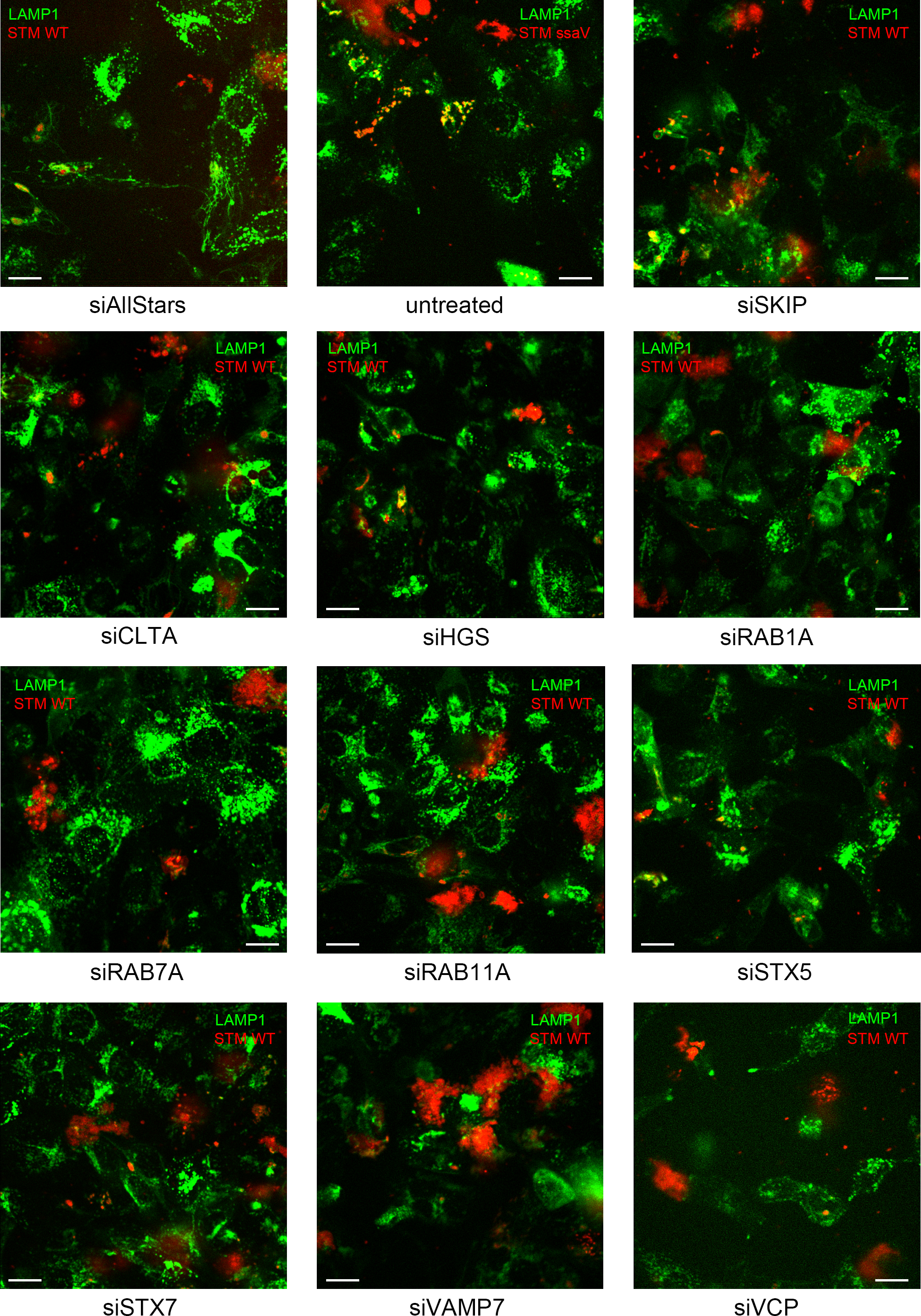

Supplement: S1 Fig — HeLa cells expressing LAMP1-GFP (green) were reverse transfected with the indicated siRNAs for 72 h (also corresponding to Fig 4). Then, cells were infected with STM WT expressing mCherry (red) at MOI = 15, and imaged by SDCM. Depicted are representative field of views 7 h p.i. Scale bar, 20 μm. (TIF) [file ppat.1008220.s007.tif]

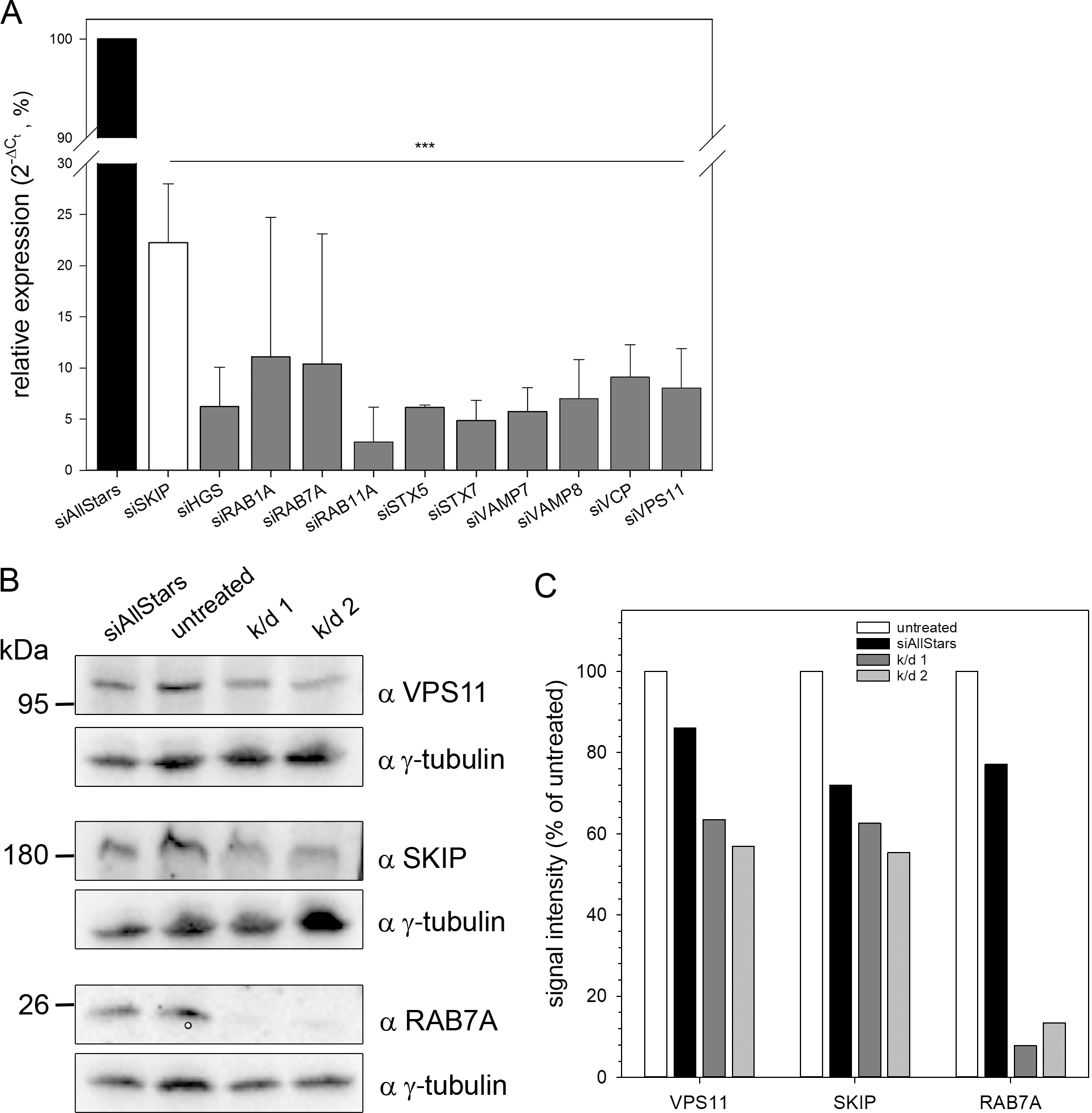

Supplement: S2 Fig — HeLa LAMP1-GFP cells were left untreated or reverse transfected with siAllStars or the indicated siRNA. A) For RT-PCR, total RNA was extracted, mRNA reverse transcribed, and the generated cDNA was used in RT-PCR. Depicted are means and standard deviation for three biological replicates (n = 3), each performed in triplicates. Statistical analysis was performed against siAllstars with Student’s t-test and indicated as: ***, p < 0.001. B) For Western blot analysis, cell lysates were processed to determine the protein levels of SKIP, RAB7A, and VPS11. Two independent knock-down assays are indicated by k/d 1 and k/d 2. As a loading control, blots were additionally processed for detection of γ-tubulin. C) Densitometry of Western blot signals for the indicated proteins. (TIF) [file ppat.1008220.s008.tif]
