# Supplementary material for: A trafficome-wide RNAi screen reveals deployment of early and late secretory host proteins and the entire late endo-/lysosomal vesicle fusion machinery by intracellular Salmonella
Source: PLoS Pathog. 2020 Jul 13;16(7):e1008220. doi: 10.1371/journal.ppat.1008220 (PMC7377517; doi:10.1371/journal.ppat.1008220)
Supplement: Attachment — Submitted filename: Response to Reviewers.pdf [file ppat.1008220.s011.pdf]

Response to reviewers' comments:

We like to thank the reviewers of our manuscript for their constructive criticisms and useful hinds. We addressed all major and minor issues raised by the reviewers. Please find our response indicated in blue font. As a general comment, we also see that the data generated in this screen offer a great potential for future work that may unravel the molecular mechanism involved. However, such detailed analyses from complex studies on their own, and we see this a future work. We started one investigation in this direction, but had to stop due to the shut-down caused by the Covid-19 pandemic. Providing the community of researchers with the results of the screen may also stimulate further studies in other labs.

Part I - Summary

Reviewer #1:
The intracellular pathogen Salmonella enterica is characterized by the formation of the Salmonella-containing vacuole (SCV) and a unique network of various Salmonella-induced tubules (SIT). The bacteria effector proteins required for the formation of SCV and SIF are well-defined; in contrast, the corresponding host proteins are poorly characterized.
In this study the authors aim to identify new host factors responsible for the formation of SCV and SIF using functional RNAi screen. They identify four different classes of host proteins: i) the late endo-/lysosomal SNARE; ii) proteins involved in the early secretory pathway; iii) proteins in the late secretory pathway, and iv) proteins involved in clathrin-coated structures. Although the study generates a large amount of data, the majority of the study is descriptive and provides limited mechanistic insights into the formation of SCV and SIF. Since the study provides only minor improvement over previous studies, such as PMID 24274083, 22701604, 25348832, 26084942, I feel that multiple major issues should be addressed before the manuscript is considered for publication.
Thank you for pointing us to these studies. Please note, that we cite of all the above-mentioned articles and also discuss the differences to our study, i.e. a focus on replication instead of SIT formation (24274083, 22701604) or a proteomic instead of a knockdown approach (25348832, 26084942), respectively. Thus, we consider the findings of this study sufficiently distinct from the other studies. However, combining all of these studies of course helps to create a holistic view of the intracellular lifestyle of *Salmonella*.

Reviewer #2:
The study is essentially a screen of the effect of the knockdown of host protein on the formation of Salmonella-containing vacuole (SCV) and Salmonella-induced filaments (SIF). 496 genes already known to be involved in protein trafficking were selected to be knocked-down by siRNA, and the functional effects were examined by using high-resolution live cell imaging to score effects on SIF induction, dynamics and morphology. Several proteins were identified to have functions in SIF formation, including the late endo-/lysosomal SNARE complex proteins (STX7, STX8, VTI1B, and VAMP7/ VAMP8), RAB7, RAB1A and RAB1B, RAB3A, RAB8A, RAB8B,VAMP2, VAMP3, VAMP4 and clathrin chains. The validation experiments included microscopy experiments, in which the select proteins containing a fluorescent tag were expressed and their interaction with SIF shown by co-localization.
While the study is potentially interesting, there are several issues, which would need to be, in my opinion, addressed prior to the publication.

Reviewer #3:

In this manuscript, a time-resolved high-content siRNA screen was performed to identify host factors involved in the dynamics of “Salmonella induced filaments”, membrane structures that are pertinent during enterocyte infection of S.Tm. The focus was on genes involved in cellular trafficking. Therefore, it has not been too surprising that a large number of the tested siRNAs showed some alterations in the Sifs. Nevertheless, the authors were able to extract very interesting data and novel links through their screen. They show the involvement of a specific SNARE complex involved in endo-lysosomal trafficking, novel roles of RabGTPases, new links with the recycling and secretion machinery and unexpected involvement of clathrin structures. The study represents an important research effort and the results are useful to the scientific community. The controls of the screen could be described in a clearer way, also showing the limitations of such screens. Showing a bit more functional analysis for one of the four proposed research directions (through the four hit groups) either through the usage of bacterial mutants, or through the application of inhibitors, or biochemical follow up would provide an important boost. Left as it is, the provided study is useful and very much appreciated by this reviewer, however it would not make the most of the performed screen.

Part II – Major Issues

Reviewer #1:

1. Since the formation of SCV and SIF are relatively latter events in the Salmonella invasion, some host factors, such those related with clathrin-coated structures, could alter the formation of SCV and SIF via impacting bacterial invasion. How do the authors distinguish host factors directly impacting the formation of SCV and SIF from the indirect modulators?

The possibility the reviewer describes is of course not excluded. However, even though we did not exactly score the bacterial invasion, in our SifScreen tool the successful infection/the presence of intracellular bacteria, i.e. invasion, was queried. Together with the ‘Notes’ section we tried to incorporate unusual phenotypes also regarding other infection parameters, such as invasion. Moreover, the problem the reviewer describes is the reason why we chose colocalization analysis as initial follow-up experiments. Even though this is of course no ultimate proof of direct interaction as the reviewer rightfully states in the second comment, but it is a first approximation, especially in conjunction with the proof of direct physical presence the proteomic studies discussed in the manuscript (see PMID 25348832 and 26084942). However, verifying direct interactions using other techniques are pursued and will be subject of future studies.

2. Colocalization cannot be used as evidence of direct or indirect interaction between two proteins (line 239). Thus, discussions related with Figures 4-7 don’t make sense to me.

The reviewer is right, that it is no direct proof. But as stated above, combining our results with proteomic studies showing the direct physical presence and the fact that many host factors that were shown in previous studies to interact with SCV/SIF appear also as hits in our screen or show colocalization at least indicates the conclusions we draw are not totally out of reach. However, to account for this uncertainty we toned down the statements we make throughout the manuscript.

3. HGS was chosen as it is the highest-ranking hit among the screen. However, knockdown of HGS did not reduce SIF formation. What is the explanation?

We observed a reduction of SIF formation by HGS k/o, albeit statistically not significant. Besides another reason, HGS might have had such an impact in screening, is due to it probably having an additional role in SCV stability, possibly ultimately affecting SIF formation. We recently could show, actually as a by-product of this screen, that the host ESCRT pathway plays a role in SCV stability (see

PMID 32017351). As HGS is a component of the ESRCT pathway, this might be an explanation.

Reviewer #2:

1. The entire study was performed in HeLa cell line, which is a cervical cancer cell line. Although this cell line has been extensively used, the authors should seek confirmation of the observation in other cell lines, ideally primary cells.

The reviewer rightfully states the wide usage of HeLa cells, which in our case applying a live cell imaging-based approach is due to their high suitability for microscopy. We fully agree that using other cell lines or primary is of immense importance, but this would require extensive efforts for parameter and condition refinement, especially in primary cells, which would in the end constitute a study on its own.

2. The co-localization experiments by using microscopy studies should be accompanied by other types of observations such as western blotting. SCV positioning experiments could also be done, such as in this article: D'Costa, V.M., Coyaud, E., Boddy, K.C. et al. BioID screen of Salmonella type 3 secreted effectors reveals host factors involved in vacuole positioning and stability during infection. Nat Microbiol 4, 2511–2522 (2019) doi:10.1038/s41564-019-0580-9. This article should also be cited and discussed.

At present, the study is largely descriptive and no new function has been claimed for the identified proteins in the SIF formation other than their upstream function.

The reviewer is right that ideally other experiments accompany the colocalization studies. We added Western blot data to support the effect of siRNA on protein levels of selected candidates. As the present work analyzed events on a single cell levels, we consider ensemble-based analyses such as Western blots of limited relevance to study the mechanisms in further detail.

Regarding SCV positioning, although our SifScreen tool in fact also queried parameters regarding the SCV, which led to a separate study on its own (see PMID 32017351), the SIF but not the SCV was the focus of the screen and the applied analysis. Moreover, even though a relevant assay, SCV positioning would not be compatible to LCI applied here due to the nucleus stain.

Besides, we were fully aware of the above-mentioned elegant study, cited and discussed it plentifully as Ref 88 in the revised manuscript, cited in lines 358, 382, 411, 428, 443. This was Ref 104 in the original manuscript that was referred to in lines 350, 375, 395, 416, 435.

Regarding other assays, such as Western blotting, i.e. protein detection, the discussion of the recent proteomic approaches studying SCV and SMM at similar time-points as analyzed here comes into play as these identify on a global scale host factors on a protein level. These studies in combination with ours lay the ground for the analysis of specific host protein interactions, which will be pursued in the future in a systematic manner, e.g. with co-immunoprecipitation.

3. The screen is performed by using an imaging system, but the timing of the picture acquisition has not been well described. Since in total 496 experiments were performed (plus controls), what was the time from experiment 1 to the last experiment? Could this timing affect the result? Moreover, the replicate number for the screen is not clearly explained. In the caption to figure 4 the replica number n=3 is included but it is not mentioned for the main screen. Also, does n=3 mean that three different wells, three different plates or three different times that the experiment was performed?

In fact, the complete screen itself, which consisted of 24 individual 96-well plates, was performed in biological triplicates, i.e. each of the 24 96-well plates was imaged three times with each replicate of 24 96-well plates containing three distinct siRNAs for each of the 496 targets (plus controls), in total 1,488 different siRNAs. We tried to specify these facts in the revised manuscript (see lines 477 ff). As mere imaging of one single plate took at least 6h (plus the infection before etc.), we could process a plate overday and one overnight. To exclude a possible influence of the timing we switched which plates were imaged overday and which overnight between replicates.

4. The screen contained the select proteins already previously known to be involved in the vesicular and membrane systems, therefore the results are not surprising and possibly not very novel either. It is still unclear how the library was designed.
It is true that the results are not surprising for host factors involved in intracellular trafficking already known to play a role in SIF biology as detailed in lines 315 ff in the discussion. These factors merely serve as another proof that our approach is feasible. However, as the definitive SIF biogenesis process regarding the host factors is still not clear, it is crucial to know exactly which host factors are participating in this process. This is especially important to know as on the long run these hitherto unknown factors might represent new targets for anti-Salmonella approaches as SIF are e.g. central for intracellular Salmonella nutrition.
As for the design of the library, as already stated above, please note that we expanded the description of the screen in lines 477 ff of the revised manuscript. We hope, it is now sufficiently clear.

5. The authors use siRNA for the screen and comment e.g. in line that loss of function might not be 100%. The only information whether the siRNA worked well is qPCR data for a subset of these proteins (11 if I counted well?). Further, it is unknown whether the protein levels were affected at the tested time point and concentration, which ultimately dictate the function. For some proteins, these siRNA conditions might need to be optimized (long half-life etc.) or even shRNA might been to be used.
Indeed, we cannot guarantee that each siRNA actually worked, but please note, that the library was not present in-house. As stated in the Material & Methods, it was provided via a platform project by an external collaboration partner, thus, we could not test each siRNA. Knockdown efficiency for largen numbers of siRNA for such screen is based on bioinformatic algorithms of the manufacturer, and confirmed by controls of the manufacturer For subsequent experiments, such as the mentioned SIF counting, we individually ordered siRNAs and could test them, therefore this seemingly random selection. However, please bear in mind, that, first, to ensure the feasibility of our approach we included our general phenotype-specific control, knockdown of SKIP, on each screening plate. Second, similar custom subgenomic libraries from the collaboration partner were executed previously (see PMIDs 20234004 and 21824245 with 471 and 473 individual targets, respectively) and the former of these studies states that all used siRNAs had at least 70% knockdown efficiency in HeLa cells on 96-well plates with 3d transfection, i.e. the same parameters we used. As 62 targets of the trafficome screen overlap with those from the mentioned screen and siRNAs from the same supplier (Qiagen) were used for our screen, it is, in our opinion, not out of reach to assume that at least a majority of the targeted host factors were also successfully silenced, even if not in all cases to this degree.
As for the protein levels, the reviewer is absolutely right, that these can be totally differently affected than the mRNAs due to half-life issues. Thus, we decided to analyze the protein levels for a selection of silenced factors from Figure 4 and provide these new data in Figure S2B. However, in Figure 4 we did not aim to reduce the SIF formation as much as possible but wanted to retest selected factors from the screen with the exact same parameters to validate the screen results for these factors. Thus, optimization of knockdown parameters seems dispensable at least for this assay.

Reviewer #3:
1. Considering the analysis pipeline, it is not entirely clear how the authors assessed the impact on invasiveness. For example, lower than 50% phenotypes (that would be hits) could also be caused by loss of invasiveness or cell death.
In fact, we did not exactly score the bacterial invasion or the cell death. Nonetheless, in our SifScreen tool the successful infection or the presence of intracellular bacteria, respectively, corresponding to invasion, as well as the cell death was queried. In case, no invasion took place or the cells died, this

was taken into consideration by assigning different levels of scores (see also lines 177 ff of the revised manuscript). Thus, together with the ‘Notes’ section we tried to incorporate unusual phenotypes also regarding these other infection parameters, even though subtle differences in e.g. invasiveness are of course not displayable like this. To make this clearer, we emphasized these aspects in the revised manuscript in lines 185 ff.

2. The majority of candidate genes showed a certain degree of interference, therefore one wonders whether this does reflect the involvement of intracellular trafficking or is there the possibility of low specificity? The authors could provide controls with another small collection of siRNA irrelevant to endocytic trafficking.
Indeed, the majority of tested host factors showed some effect. However, even if most of the target genes of the trafficome are trafficking-related as defined per GO terms, there is also a considerable proportion of target genes also or only involved in trafficking-unrelated processes. One good example are targets that are also or only involved in metabolic processes (23% of the trafficome are annotated to GO:000815/metabolic process). Indirect effects, especially in the lower hit range, are likely to take place with these targets, at least with several of them. Thus, we think it is not a problem of low specifity but of indirect vs. direct effects. Moreover, effects on the general fitness of the cells can likewise not completely be excluded. Even though we queried the cell health on a global level with our SifScreen tool, subcellular detrimental effects that might impact as a by-product SIF biogenesis cannot fully be eliminated. Therefore, we decided to focus on mid- and high-ranging hits for the follow-up experiments.

3. A number of candidates have been implicated, and described in S.Tm. intracellular trafficking. Therefore, it would be great to have a bit more follow up- do they act in the same pathway? The authors provide 4 directions, however with very little functional follow up or confirmation through alternative approaches, such as inhibitors. Another good addition would be a link with S.Tm effectors, for example the authors could test this through a verification of SseF and SseG’s role in the recruitment of screened factors.
The reviewer is right that more functional elucidation is desirable. Thus, both of the reviewer’s suggestion, the application of inhibitors and STM mutants, are much appreciated. Therefore, we started to elucidate how RAB1 is recruited to SCV/SIF. Two scenarios are possible: either via the early secretory system and COPI vesicles as suggested by the screen results overall with *Coxiella burnetii* being an example of another intracellular pathogen where such recruitment takes place (see PMID 20937765); or directly from the cytosol with *Yersinia pestis* being an example for this possibility (see PMID 26495854). To test this, we first applied two Golgi inhibitors, brefeldin A (BFA)and golgicide A (GCA) (which would allow the differentiation exactly which ARF1 GEF might be involved) to disturb the early secretory system. The resulting dispersal of the Golgi was indeed successful in our hands (see the see the **accompanying Figure 1B** and **C** for BFA and GCA, respectively, in comparison to untreated cells in **A**). Moreover, pulse-chasing STM-infected HeLa cells from 5-6 h p.i. with BFA did not disturb the formation of SIFs (see **accompanying Figure 1D**), so that analysis of potential host factor colocalization is still feasible. This would have been followed up by next investigating the localization of RAB1B WT as well as dominant-negative and dominant-active mutants in an STM infection setting under BFA/GCA conditions. This would have required the thorough testing of different parameters, such as which time points p.i. are relevant to analyze. Then, this could have been combined with testing STM effectors mutants known to be involved in SCV/SIF formation, i.e. PipB2, SifA, SseF, SseG, SseJ, and SopD2.
However, much to our deepest chagrin, we were not able to follow up on these experiments as our laboratory was effectively shut-down due to the Corona pandemic.

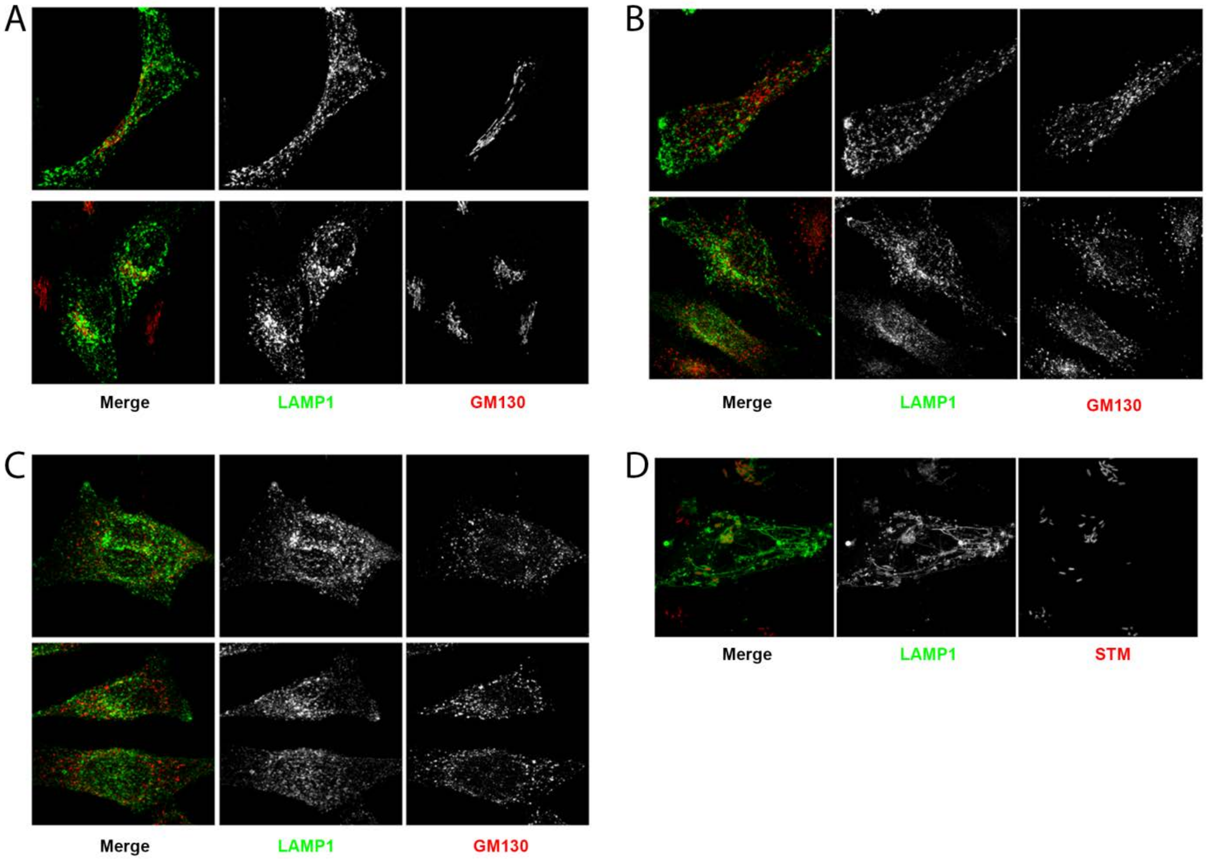

Figure 1. Analysis of effect of BFA and GCA on the Golgi network of HeLa cells. (**A-C**) HeLa LAMP1-GFP cells were left untreated, (**A**) or pulse-chased for 1 h with 10 µM BFA (**B**) or GCA (**C**), respectively. Then, cells were fixed and stained for GM130. Shown are two representative cells per condition. (**D**) HeLa LAMP1-GFP cells were infected with mCherry-labeled STM and pulse-chased for 1 h with 10 µM BFA at 5 h p.i. Imaging was performed using CLSM.

4. Through the hits, the authors propose a diverse origin of host membranes for the Sifs, however this lacks solid evidence. Therefore, it should be down-toned a bit.
As suggested by the reviewer, we went through the manuscript and tried to overall tone down this aspect. Please note that the model of heterogenous origin of membrane material in SIF as supported by proteomic analyses.

5. It would help if the authors could be more structured about the points they want to make in the discussion. It appeared a bit as a mini review.
Our intention was first to generally set our results in context to previous results, especially in comparison to proteomic approaches. The next paragraphs were meant to discuss the four categories of hits which we also defined in the abstract. However, it is true that the order in the discussion did not match the one in the abstract. Thus, we reorganized the discussion and tried to tighten it in doing so.

Part III – Minor Issues: Editorial and Data Presentation Modifications

Reviewer #1:
1. The microscopy images shown are often very small, making it difficult to determine subcellular localization.

We increased size and detail magnification in Fig. 1D. The micrographs are generated with high resolution that should be well visible in the online vision of this work, once published

2. Please check grammar throughout the manuscript.

Spelling and grammar were both carefully checked throughout the revised manuscript.

Reviewer #2:

1. Line 526: Was the OD600 monitored for the bacterial cultures?
Yes, indeed the OD600 was monitored for the subcultures of each and every individual plate experiment. We added the range of the corresponding values in lines **XY** of the revised manuscript.

2. Line 560 "Quantification of SIF formation" – the paragraph does not contain sufficient description.
We are sorry that this paragraph lacked information in the reviewer's opinion. We expanded this paragraph (lines 602 in the revised manuscript) and hope that it now delivers sufficient description.

3. Table 1 – the scoring cutoffs should be provided with the reference where to find information about the scoring. Uniprot accession numbers should be introduced (particularly as Uniprot is used as the localization information source) as well as references to the source of information for localization apart from Uniprot.
Again, we hope to provide sufficient clarification by the following alterations/additions: we have added a mention where to find more information on the scoring. Furthermore, we added in Table S1 UniProt information including direct hyperlinks and refer to it in the caption, as we find the information better suited in Table S1 instead of Table 1 for reasons of clarity. Moreover, please note, that we did not use any other source for localization information apart from UniProt, thus, the mentioning of UniProt in the localization footnote suffices in our opinion.

4. Table 2. The description should be improved. Uniprot on NCBI accession numbers should be referenced.
As mentioned above for Table 1, information regarding host factors, also encompassing those of Table 2, are given or were added to Table S1. We modified the caption to make it clearer, but again, for reasons of clarity, we refer to Table S1 in the caption for this additional information.

5. Line 1106: Mention that these are date from the current study.
We are sorry that this was ambiguous, but actually this statement des not refer to our study but to PMID 25348832, i.e. the corresponding proteome study. Thus, we included this reference in this footnote to make that clearer.

6. Line 1120-1121: Mention time after transfection.
As suggested we added this information in the caption.

7. Figure 1. Figure D is too small in proportion to Figs. A- C, especially C.

We revised Fig. 1 and increased the size of panel D to maximal width. The magnification of the detail insets was increased 200 %.

8. Figure 2. The information about the time(s) of infection and MOI are needed.
The missing information were added in the figure itself and in the caption.

9. Figure 4. How many cells were counted per image?
As it was counted with a 40x objective usually 10-15 cells were present in one field of view. Due to the high MOI of 50 most cells were infected, so that roughly 10 cells per field of view were actually counted resulting in 10 analyzed fields of view per biological replicate.

10. Figure 5. It is left unclear why in certain cases cells were co-transfected with LAMP1 by using transiently expressing LAMP1 and in other cases stable transfection was chosen.
Ideally we would have liked the experimental setup to be identical in all cases. However, for the transfection constructs for the colocalization analysis, we relied on different sources, i.e. AddGene, , own constructs using DNASU or personal gifts. Thus, regarding the choice of fluorescent proteins attached to the different structures we were bound by the available material. In case we could analyze LAMP1 tagged with GFP we applied a stably transfected cell line we successfully use for years in our laboratory (see PMID 25254663). Unfortunately, we do not possess a corresponding cell line for LAMP1 with a red fluorescent protein, which forced us to use transient transfection. Nonetheless, as both approaches were successfully used for colocalization experiments in *Salmonella* (see e.g. PMID 25348832), we consider this fact not relevant for the outcome of the experiments.

11. Table S2 and S3. Improve the description of the table (Caption) to increase the clarity.
Regarding Table S2, we think lack of clarity originated from uploading not the newest version of Table S2, which did not fully correspond to the caption. We apologize for that and included for the revision the correct file for Table S2. Nonetheless, we also revised the captions for both Table S2 and S3 to make it clearer.

12. Line 1199: What is special plastic vs standard plastic? Unclear.
We added the word proprietary to make clear, that it is a special type of plastic with proprietary features only the company Corning supplies in this fashion.

13. Appears that there are only 11 targets for q PCR but over 400 genes we initially knocked down.
Please refer to the answer to the reviewer's ‚Major Point #5' above as to why we did not test each siRNA applied.

14. Figure 2. How long did the phenotypic screening take? Was that considered while interpreting the changes in time?
As now added to the figure and the caption, the screening itself took place 1-7h p.i. And yes, indeed, we took the whole time of screening into account, since the scoring was performed by analyzing time-lapse movies of the individual plate positions. In fact, it even facilitated scoring as formation of SIF was more readily detectable this way. We made this clearer in the description of the screen analysis in lines 185 ff of the revised manuscript.

15. More images could be made available for the protein targets, whose knockdown affects the SIF formation. The attached tables are not very informative.
As the reviewer suggested we provide a selection of image directly from the screen corresponding to the targets from Figure 4 in the new Figure S1.

16. The article should be re-written in a more clear manner. There are issues throughout with the sentence clarity, for instance the authors often use undefined pronouns in the beginning of the 185sentence, which makes it uneasy for the reader to follow the meaning (e.g. sentence in lines 90, 179, 184, 257, 272, and others).

We rephrased the text as suggested and removed undefined pronouns

Examples of other unclear sentences:
Line 121-122 “a connection to clathrin-coated structures.” Unclear. Connection of what to what?
Rephrased, line 121-122

Line 279-280: “this screen identified AP2A1 as being present on SMM/SCV or scoring high-ranking (Table S2), respectively.” Unclear.
Rephrased, lines 302 ff.

Line 283-284: “Accordingly, the main coat determinants implicated in the formation of CCVs were among the high-scoring hits, including both clathrin light chains.” What do authors mean by coat determinants?

Rephrased, lines 306 ff.

Line 311: “Proteomics shown presence or absence of host factors on the organelle of Interest (…)”. I would rephrase this to be “Proteomic analyses lead to the…”and cite the literature.

Changed as suggested, lines 335 ff.

Line 315-316 “However, a functional role revealed by RNAi does not necessarily require colocalization of the host factor with the compartment, because a function may be mediated indirectly, involving several interacting partners.“ What function? Which compartment? The sentence is overall unclear.

The section was rephrased and more specifically related to endosomal remodelling. Lines 338 ff.

Line 360-361: “It is also a RAB1B effector and COPI and COPII tether [97, 115, 116], partly in conjunction with COG [117], besides being likewise able to bind STX5 [118].“ Rephrase this sentence.

This section was rephrased, lines 387 ff.

Non grammatical.
Line 426-427: “It is peculiar that proteomics, as well as our screen, indicate an involvement of the AP-2 complex, but one of the other adaptor complexes. This is noteworthy…” Both sentences are unclear. First sentence does not specify which proteomics experiment is referred to, and it is also unclear what the involvement of AP-2 might be – in what? The second part of the sentence is not grammatical. The second sentence starts with the undefined pronoun, which is highly discouraged and introduces lack of clarity.
This section was rephrased and citations were added. Lines 436 ff.

We went through the whole manuscript and rewrote not only the unclear sentences the reviewer mentioned but tried to improve the clarity overall.

17. The abstract, instead of providing points, maybe make could be re-written in a more exciting way.
Thank you for this suggestion. We tried a couple of alternatives, yet think it is important to list the main results (hits) of this screen. We emphasized in the abstract what is new and remarkable.

18. The supplementary text:” Considerations for screen design and setup” should be rewritten in a more concise and clear manner. Also, pay attention to the use of undefined pronouns.
As with the main text, we went through the whole supplementary text and tried to increase the comprehensibility overall.

Reviewer #3:
1. Undefined abbreviations: LCI, AAA, VCP etc.
Live cell imaging (LCI) is defined in line 116 in the revised manuscript. For AAA, we included the definition. Besides, we went through the manuscript to identify unexplained abbreviations and found no further ones. VCP as all other host factors mentioned is not an abbreviation per se but the official gene symbol. Please, refer to Table S1 for the full name. Otherwise we would need to explain each host factor we mention in the main text which would considerably blow up the manuscript.

2. Choice of wordings:
Wording line 226: "exactly quantified"

Rephrased, line 245 in the revised manuscript

Wording line 294: "out data"
corrected, line 316 in revised manuscript
